# The Role of Gut, Vaginal, and Urinary Microbiome in Urinary Tract Infections: From Bench to Bedside

**DOI:** 10.3390/diagnostics11010007

**Published:** 2020-12-22

**Authors:** Tomislav Meštrović, Mario Matijašić, Mihaela Perić, Hana Čipčić Paljetak, Anja Barešić, Donatella Verbanac

**Affiliations:** 1 University Centre Varaždin, University North, 42 000 Varaždin, Croatia; tmestrovic@unin.hr; 2 Clinical Microbiology and Parasitology Unit, Dr. Zora Profozić Polyclinic, 10 000 Zagreb, Croatia; 3 Center for Translational and Clinical Research, University of Zagreb School of Medicine, 10 000 Zagreb, Croatia; mihaela.peric@mef.hr (M.P.); hana.paljetak@mef.hr (H.Č.P.); 4 Division of Electronics, Ruđer Bošković Institute, 10 000 Zagreb, Croatia; anja.baresic@irb.hr; 5 Department of Medical Biochemistry and Haematology, University of Zagreb Faculty of Pharmacy and Biochemistry, 10 000 Zagreb, Croatia; donatella.verbanac@pharma.unizg.hr

**Keywords:** microbiome, microbiota, gut microbiome, vaginal microbiome, urobiome, urinary tract, urinary tract infection, UTI, bacteriuria, dysbiosis

## Abstract

The current paradigm of urinary tract infection (UTI) pathogenesis takes into account the contamination of the periurethral space by specific uropathogens residing in the gut, which is followed by urethral colonization and pathogen ascension to the urinary bladder. Consequently, studying the relationship between gut microbiota and the subsequent development of bacteriuria and UTI represents an important field of research. However, the well-established diagnostic and therapeutic paradigm for urinary tract infections (UTIs) has come into question with the discovery of a multifaceted, symbiotic microbiome in the healthy urogenital tract. More specifically, emerging data suggest that vaginal dysbiosis may result in *Escherichia coli* colonization and prompt recurrent UTIs, while urinary microbiome perturbations may precede the development of UTIs and other pathologic conditions of the urinary system. The question is whether these findings can be exploited for risk reduction and treatment purposes. This review aimed to appraise the three aforementioned specific microbiomes regarding their potential influence on UTI development by focusing on the recent studies in the field and assessing the potential linkages between these different niches, as well as evaluating the state of translational research for novel therapeutic and preventative approaches.

## 1. Introduction

During evolution, microorganisms developed intimate relationships with humans by colonizing various body environments at the interface with the outer part of the body and invaginations such as the skin, nose, mouth, gut, vagina, and urogenital tract—constituting in turn an integrated metaorganism [1,2]. The result is a reciprocal adaptation and functional consolidation which confers substantial advantages to humans and their colonizers (primarily bacteria). Furthermore, our immune system has co-evolved with the resident microbiota and given rise to complex mechanisms for recognizing and destroying invading microorganisms while preserving its own bacterial species [2,3]. There has been a myriad of studies over the last decades exploring the role of gut microbiota in health and disease, followed by studies of other high-volume microbiome organs such as the vagina and the skin; however, fewer reports exist on the role of the microbiota at other body sites such as the urinary tract [4].

Urinary tract infections (UTIs) belong among the most common bacterial infections, affecting approximately 150 million people globally every year [5]. With incidence increasing with age, and a lifetime incidence of 50–60% in adult women [6], UTIs are a significant burden on society and the healthcare system, not in the least due to treatments that contribute significantly to antibiotic resistance [7,8]. These infections are caused by a wide range of pathogens, and the underlying pathogenesis is usually explained by the ascending of intestinal bacteria; however, recent studies have implied the significant roles of vaginal, intestinal, and urinary microbiota in the regulation of disease activity. This review aims to appraise these three specific microbiomes regarding their potential influence on urinary tract infection (UTI) development by focusing on the latest studies in the field from the clinical milieu, and assessing the potential linkages between different niches, as well as evaluating the potential of introducing certain prophylactic/therapeutic measures based on the current insights.

## 2. The Unknowns and Open Questions in the Era of Metagenomics

**Figure 1:**
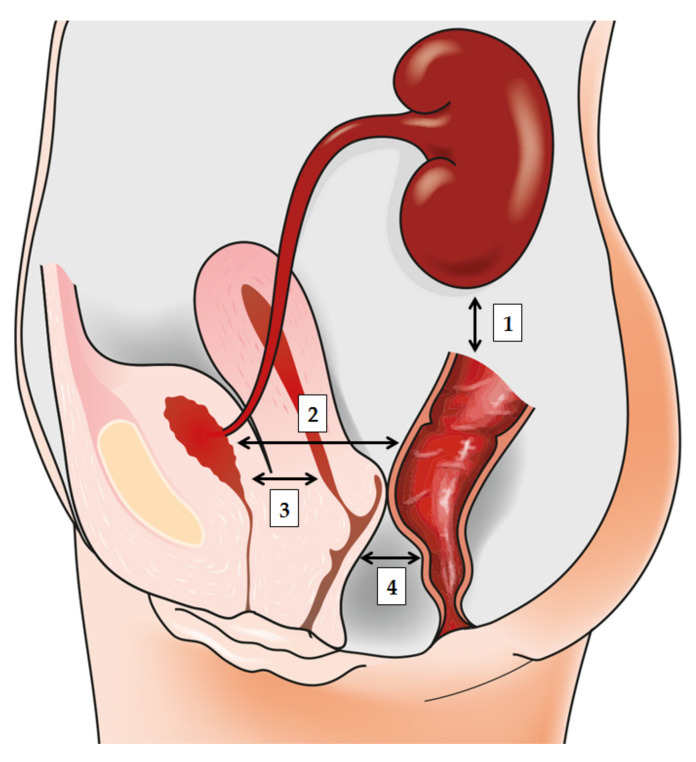
Bidirectionally informed communication between the gut, urinary tract, and genital tract: (1) gut–kidney axis that plays a role in various renal disorders; (2) gut–bladder axis and the link between the “intestinal bloom of uropathogens” and UTI development; (3) vagina–bladder axis where vaginal dysbiosis may prompt UTI development acting as a reservoir for *Escherichia coli* or prompting “covert pathogenesis”; (4) gut–vagina axis where intestinal dysbiosis may influence the local vaginal milieu.

The origin of a majority of bacterial UTIs is presumed to be in the gut [3], hence rendering it a natural first step in the investigation of the relationship between intestinal microbiota and subsequent development of bacteriuria and UTI. Recently, the well-established diagnostic and therapeutic paradigm for UTIs has come into question with the revelation of a multifaceted, symbiotic microbiome in the healthy urogenital tract [9,10]. In women, vaginal bacteria play a pivotal role in the pathogenesis of UTIs, while the gut microbiota are the ultimate source of bacterial strains responsible for cystitis and pyelonephritis in most of the cases, clearly demonstrating the crosstalk and interconnectedness of these two niches [11] (Figure 1). Therefore, recognizing factors that affect both gut and vaginal microbiota is indispensable for understanding the pathogenesis of UTI, and designing interventions to prevent it.

With the advent of affordable sequencing techniques, metagenomic approaches independent of culture have shined a more detailed light into the bacterial diversity of urinary microbiota. Since urine has been traditionally seen as naturally sterile [12,13] due to a plethora of methodological biases, novel techniques have moved away from the consideration of only dominant bacteria from rapidly and aerobically cultured urinary specimens [14]. Likewise, the higher frequency of UTIs in women than in men has prompted considerations that the source of bladder colonization is genital as a result of the small size of the female urethra [14,15]. This resulted in a hypothesis that the bladder microbiota is of vaginal origin (apart from UTIs), overlooking the fact that men also have UTIs and a urinary microbiota [16]. But how do UTIs link to gut microbiota, as opposed to vaginal ones?

Finally, there is no consensus on a strict definition of a UTI [17], as the bacterial colony-forming unit-based thresholds that delineate infection by standard clinical urine culture are still a matter of debate. This ambiguity is further complicated by the discovery that urinary bacteria and, thus, “bacteriuria” can be found in the urine of almost every individual—including those that do not present with urinary symptoms [18] and the effect has to therefore be refined beyond the mere presence/absence of certain taxa. As asymptomatic bacteriuria has even been viewed as a protective parameter against recurrent UTI [19], our improved understanding of the urinary microbiome may suggest mechanisms for this clinical observation and inform further translational endeavors. 

## 3. The Importance of Gut Microbiota in Urinary Tract Infections

It is well-known that the composition of gut microbiota can influence the health of distant body organs [20]. The majority of studies in the past decade have focused on characterizing the complex interactions in the microbiota–gut–brain axis, which are crucial for the maintenance of human psychological and physiological wellbeing and associated with various neuropsychiatric disorders [21,22,23,24]. Similar to the gut and the brain, a bidirectional relationship also exists between the gut and the kidney. Mounting evidence indicates that gut microbiota plays an important role in the gut–kidney axis [25] with the dysbiosis of the gut microbial community implicated in the pathogenesis of various renal disorders, thus indirectly contributing to hypertension and chronic kidney disease [26], as well as urinary stone disease [27] (Figure 1). A more direct link between gut microbiota dysbiosis and the urinary system is evident in urinary tract infections.

The pathogenesis of the UTI typically starts with contamination of the periurethral space by uropathogens residing in the gut, followed by colonization of the urethra and ascending migration to the bladder [28]. UTIs are predominantly caused by uropathogenic *Escherichia coli* (UPEC), which is responsible for over 80% of community-acquired infections, while healthcare-related infections are associated with *Staphylococcus*, *Klebsiella*, *Enterobacter*, *Proteus*, and *Enterococcus* [28,29]. UPEC strains are found in abundance in the gut of patients with UTIs and are thus considered to originate from the gut [30,31]. UPEC differs from commensal *E. coli* by possessing extragenetic material encoding for genes involved in bacterial pathogenicity, i.e., adhesins, toxins, surface polysaccharides, flagella, and iron-acquisition factors [32,33]. UTIs are more prevalent in women; the female urethra is closer to the anus and shorter than the male urethra, facilitating the migration to and colonization of gut microorganisms in the urinary tract [15,34].

Breastfeeding was reported to have a protective effect against UTI in infants and preterm neonates, additionally supporting the hypothesis that the origin of UTI is in the gut [35,36]. Furthermore, Paalanne et al. noted several differences at the family and genus levels, most importantly the higher abundance of *Enterobacter* in the gut microbiota of pediatric patients with UTIs when compared to healthy controls, suggesting the intestinal environment and its microbial community is associated with the risk of UTI in children [37].

A recent study from Magruder et al. further described the gut microbiota–UTI axis [38]. The authors demonstrated that the increased abundance of *E. coli* in the gut was associated with future development of *E. coli* bacteriuria and *E. coli*-induced UTI. Also, the *E. coli* strains in the gut displayed greater resemblance to the *E. coli* strain in the urine from the same subject, thus supporting the hypothesis that gut microbiota is a source of urinary tract colonization and UTI [38].

Likewise, by combining semiquantitative culturing with comparative genomics, Thänert et al. provided evidence for the repeated transmission of uropathogens between the gut reservoir and the urinary tract, showing that recurrent UTIs (rUTIs) are habitually preceded by a so-called “intestinal bloom of uropathogens” [39]. The provided data expand our knowledge of the temporal dynamics of pathogen clearance and persistence following symptomatic UTI, but also underline the significance of acknowledging intestinal colonization with antimicrobial-resistant microorganisms as inherent to the pathophysiology of rUTIs [39].

Finally, a study by Magruder et al. from 2020 demonstrated that high relative abundances of two bacterial taxa—*Faecalibacterium* and *Romboutsia*—can be linked to the decreased risk for *Enterobacteriaceae* bacteriuria and UTI in kidney transplant recipients [40]. This research group additionally reported an inverse relationship of the relative abundances of the aforementioned two taxa with the relative abundance of *Enterobacteriaceae*, lending further support for a growing notion that intestinal commensal organisms are related with lower risk of infectious complications, which is already deep-rooted for *Clostridioides difficile* infections [40].

## 4. The Vaginal Microbiome at the Intersection of Health and Disease

The vaginal microbiota in women of reproductive age is dominated by several *Lactobacillus* species, which includes *L. crispatus*, *L. jensenii*, *L. gasseri,* and *L. iners* [41,42,43]. These lactobacilli maintain the vagina’s characteristic low pH (primarily by producing lactic acid) and produce antimicrobial compounds such as hydrogen peroxide and bacteriocins [41]. However, while a *Lactobacillus*-dominant vaginal microbiome is seen as normal or “healthy”, many women of reproductive age harbor a much more diverse vaginal microflora without lactobacilli (or low levels of these bacteria); more specifically, they are colonized with a mixture of Gram-negative anaerobic organisms, Actinobacteria, and other Firmicutes [44]. In certain instances, this community state, termed type IV, can lead to dysbiosis known as bacterial vaginosis (BV) [45].

Studies of vaginal microbiota have long indicated that the presence of certain bacterial species and other microbial characteristics can be linked to the disease-free state of the genitourinary tract [46,47]; however, mounting evidence shows that the vagina can harbor uropathogenic bacteria. First and foremost, the vagina can act as a reservoir for *E. coli*, with multiple studies showing that women with a history of rUTIs more commonly have *E. coli* in their vaginal introitus or vagina in comparison to healthy controls [43,48]. Recently, Brannon et al. demonstrated that diverse urinary *E. coli* isolates not only adhere to but also invade vaginal cells in acute and chronic murine UTI models, highlighting the ability of *E. coli* to reside in the vagina after UTI [49]. Conversely, their vaginal colonization model indicated that vaginal colonization can subsequently seed the urinary bladder with the pathogen and prompt rUTIs. These findings demonstrate the ability of *E. coli* to establish a vaginal intracellular reservoir where the pathogen is safe from extracellular stressors before it causes an ascending infection [49].

This is where the protective role of vaginal lactobacilli comes into play, by preventing colonization with *E. coli* and other potential uropathogens in the first place. Studies have repeatedly shown that women with low levels of lactobacilli are more commonly colonized with vaginal *E. coli* than those with lactobacilli-dominated microbiomes, which naturally decrease the risk of UTI development [43,50]. Recent studies confirm that many species of lactobacilli (most notably *Lactobacillus crispatus*) have the propensity to inhibit *E. coli* growth, likely through creating and maintaining a low pH environment [51,52]. Furthermore, multiple studies in both non-pregnant and pregnant women show that bacterial vaginosis (characterized by the decrease of protective lactobacilli) increases the risk for colonization of vaginal introitus and UTI development [53,54,55].

Several vaginal bacterial species are often detected after urine culture but are underappreciated as uropathogens, while other vaginal species can be under-reported as a result of their fastidious nature. One notable example is *Gardnerella vaginalis*, which is implicated in rUTIs and kidney disease, but also in systemic infections originating in the urogenital tract [43]. One proof-of-concept study has shown that *G. vaginalis* bacteriuria is significantly linked to patients with a history of rUTI or ongoing pyelonephritis, most of them with pyuria and clinical symptoms [56]. Klein et al. recently showed that the use of total laboratory automation can increase the yield of *G. vaginalis* and other previously underestimated constituents of vaginal microflora in urinary samples, highlighting their potentially relevant role in the development of UTIs [57].

Furthermore, certain vaginal bacteria that are not widely considered as uropathogens can be briefly present in the urinary tract, perturb host-pathogen interactions, and prompt injury or immunomodulation, which is referred to as “covert pathogenesis” [43]. Several studies addressed this hypothesis in mouse models. More specifically, the presence of *Streptococcus agalactiae* (or group B *streptococcus*) was shown to aid *E. coli* survival within the bladder during the early hours of acute infection despite its rapid clearance by the host [58], while the other study demonstrated a similar effect of *G. vaginalis*, which also acted as a trigger of rUTIs [59].

Finally, hormones in women may play a substantial role in changing the vaginal microbiome. The loss of estrogen during menopause can decrease the relative amounts of *Lactobacillus* species, with a subsequent rise of UTI rates and the development of rUTIs [47,60]. This is seen as a hallmark of the genitourinary syndrome of menopause, characterized by vaginal epithelium thinning and vulvovaginal atrophy, as well as the aforementioned loss of lactobacilli within the vaginal microbiome [61]. Along those lines, there was a pivotal study by Pabich et al. on 463 postmenopausal women, where colonization with *E. coli* was more frequently observed in women without estrogen replacement and inversely correlated to the presence of *Lactobacillus*; moreover, the disturbances of vaginal microbiota were a predisposing factor for rUTIs [62].

## 5. The Increasingly Appreciated Significance of the Urinary Microbiome

Pervasive implementation of techniques designed to identify all microorganisms in the urine, most notably sequencing-based techniques (e.g., 16S rRNA sequencing or whole genome sequencing), but also cultivation-based methods of expanded quantitative urine culture [13,63], has shown that a wide range of bacterial species can be found in the urine of healthy, asymptomatic individuals [64,65]. The analysis of the urinary microbiome as a field of research is still in its infancy; hence, there is much to learn and discover regarding its role in maintaining urinary bladder and urinary tract homeostasis in different contexts. Nonetheless, emerging data suggest that the urinary microbiome may indeed play a significant role in a myriad of pathologic conditions of the urinary system, such as urge incontinence [66], overactive bladder [67], and urinary bladder cancer [68]. It is then no wonder that the urinary microbiome is also implicated in the pathogenesis of UTIs.

One pivotal study demonstrated that patients who developed UTIs during a trial of bacterial interference presented with a lower diversity of the urinary microbiome in comparison to those who did not develop UTIs [69]. This was corroborated in a study by Bossa et al., who demonstrated that urinary microbiome changes preceded the development of a UTI, and that the urinary microbiome basically normalized after treatment—suggesting its relative stability over time [70]. In conclusion, perturbations in the urinary microbiome (a condition referred to as dysbiosis) may suggest the upcoming development of a UTI, thus prompting the view of UTI development as “urinary tract dysbiosis” [71]. Naturally, further longitudinal studies of the urinary microbiome in patients with different types of UTIs and pathological conditions are needed for resolute conclusions.

More acutely, there is a need to answer the question of what should be considered a normal human urinary tract bacterial repertoire. A recent study by Morand et al. revealed the breakdown of taxa percentage in the main phyla of the human urinary tract into: Proteobacteria (35.6%), Firmicutes (31.3%), Actinobacteria (22.4%), Bacteroidetes (6.4%), and others (4.3%) [72]. The authors also concluded that the majority of pathogenic bacteria are constituents of the commensal human urinary tract bacteria, and their pathogenicity occurs due to imbalance in their relative abundances [72].

Moreover, in a recent approach to decipher the urinary microbiota repertoire with the use of culturomics, a research group from France tested 435 urine samples and isolated a total of 450 diverse bacterial species—including 256 previously not found in urine and 18 completely new species [73]. This study not only increased the known urinary microbiome repertoire by 39%, but it has also shown that 64.1% of bacterial species were previously isolated from gut microbiota, compared to only 31.7% previously found in the vagina. As a result, this study calls for a paradigm shift to look at the urinary microbiota as mostly derived from the intestinal tract [73], but more research is needed to confirm and ascertain this point-of-origin hypothesis. 

Grine et al. studied the potential implications of archaea in the context of the urinary microbiome, and pinpointed *Methanobrevibacter smithii* as the putative species with a potential role in community-acquired UTI (in association with enteric bacteria) [74]. In their paper, this methanogen was detected in 9% of urine samples in two unrelated samples of patients. Previous metagenomics and culture-based studies have missed this species and other methanogenic archaea, which are otherwise detected (and often cultured) from the intestinal and oral microbiota [74]. This is definitely a nascent field of study, especially considering the potential metabolic cooperation between enterobacteria that produce hydrogen, and methanogens that use it as a major substrate.

A recent culture-independent analysis of patients refractory to standard antimuscarinic therapy for recurrent UTIs revealed the existence of a diverse urinary microbiota, hinting that persistent bladder colonization might provoke the pathology of their chronic condition [75]. The most common urotype was dominated by *Corynebacteriaceae*, found in 31.58% of patients included in the study. The authors have also noted that approximately 40% of the urine specimens examined by routine laboratory approach were reported as “mixed growth”, suggesting that the focus on reporting a single dominant organism might be revisited in patients with specific conditions who suffer from recurrent UTIs [75].

By analyzing urogynecologic surgical patients, Thomas-White et al. have demonstrated that the risk of post-operative UTI can be linked to the composition of the day-of-surgery microbiome of catheterized urine—suggesting a possible, previously underappreciated etiology for the urinary microbiome in this condition, which can subsequently open the door for tailored preventative measures [76]. More specifically, the risk of post-operative UTI was primarily associated with the depletion of certain *Lactobacillus* species, most notably *Lactobacillus iners*, but also *Peptoniphilus* spp. [76]. The pertinent question is: do these observations open the door for potential prophylactic and/or therapeutic approaches?

## 6. Applying Novel Insights to Clinical Practice: Can We Reduce the UTI Risk?

Although antibiotics remain the commonly recommended treatment for UTIs, the long-term alteration of normal intestinal microbiota and emergence of multidrug-resistant microorganisms has made the advancement of alternative therapeutic options for combating the infection a necessity [77]. Along with the efforts in developing precision antimicrobial therapeutics and UTI vaccines (extensively reviewed in Spaulding et al. [78] and O’Brien et al. [79]), gut microbiota dysbiosis was recognized as one of the key contributing factors to developing UTIs, and modulating the gut microbial community could prove a promising strategy in disease prevention and treatment. The use of commensal bacteria as probiotics was demonstrated to reduce the abundance of pathogens, restoring the microbiota homeostasis [80]. Koradia et al. reported that the administration of a commercially available probiotic product containing two strains of lactobacilli supplemented with cranberry extract significantly lowered the number of recurrent UTIs in premenopausal women when compared to a placebo in a randomized, double-blind, placebo-controlled pilot study [81]. A similar study demonstrated a probiotic mixture was more effective than the placebo at reducing the risk of recurrent UTIs in children after their first episode of febrile UTI [82]. A noninferiority randomized controlled trial in postmenopausal women with recurrent UTIs confirmed oral probiotics (*Lactobacillus rhamnosus* GR-1 and *Lactobacillus reuteri* RC-14) did not meet the noninferiority criteria in the prevention of UTIs when compared to antibiotic treatment [83]. Unlike antibiotic treatment however, the use of lactobacilli did not increase antibiotic resistance [83].

Recently, a very interesting link between fecal microbiota transplantation (FMT) and recurrent UTI was found, further confirming the gut microbiota–UTI axis and revealing a new potential treatment option for the disease. Tariq et al. reported a reduced UTI frequency after FMT for the treatment of recurrent *Clostridioides difficile* infection (rCDI) [84]. The study noted an improved antimicrobial susceptibility profile in microbial isolates from urine samples after FMT, suggesting gut decolonization of multidrug-resistant organisms in favor of less pathogenic and commensal microbes [84]. Several case reports confirmed beneficial effects of FMT for patients with rCDI or IBS who concomitantly suffered from rUTIs, demonstrating reduced or no UTI recurrences after the FMT procedure [85,86]. Moreover, two recent studies provided insight on the application of FMT for the treatment of rUTI in kidney-transplant recipient patients without concomitant disease [87,88]. Both author groups report a marked decrease or absence of UTI-inducing bacterial strains in the urine samples after FMT, and no symptoms of subsequent UTIs in a 12-month follow-up period, suggesting successful modification of the urinary microbiota by FMT and an important role of the gut microbiota composition in the pathogenesis of rUTIs [87,88]. A recent case report along those lines showed that the disappearance of UTIs in a patient presenting with recurrent infections of the urinary tract resulted from the reduction of *Enterobacteriaceae* in the gut microbiota (from 74% to 0.07%) after FMT [89]. A clinical trial determining the effectiveness of fecal transplantation in the treatment of refractory, recurrent urinary tract infections (NCT03050515) is currently underway, assessing change in frequency of UTIs following FMT as well as the efficacy of FMT in modifying the UTI bacterial profile in urine samples to commensal, pan-sensitive organisms.

The mentioned interactions between the constituents of the vaginal microbiome and the risks of UTI development may have clinical implications regarding treatment and prevention choices. In theory, the use of oral or intravaginal probiotics should restore protective vaginal lactobacilli, halt the development of UTIs, and consequently reduce the use of antimicrobials as a preventive strategy [47]. Nonetheless, data on this type of approach are very limited and available study results are thus far conflicting [90]. A small, randomized, placebo-controlled, double-blind phase 2 trial has shown that *L. crispatus* probiotic, directly administered to the vagina in a suppository form, may decrease rates of recurrent UTI in comparison to a placebo [54]. Still, it has to be noted that a recent Cochrane review of all probiotic approaches to UTI prevention found insufficient evidence for this type of approach, with only a handful of studies appropriate for inclusion [91]. Therefore, more randomized, double-blind, placebo-controlled studies with appropriate sample sizes, diligently tailored protocols, and a carefully selected probiotic strain are definitely needed for steadfast conclusions regarding this approach.

Many postmenopausal women may be offered topical hormone therapies for primary or secondary prevention of UTI [47,90]. A systematic review has reported that all commercially available vaginal estrogens have utility in patients with urinary urgency, frequency, or nocturia, as well as recurrent UTIs [92]. However, a hormonal treatment is not the only option; a recent study by Sarmento et al. showed a statistically significant increase in the percentage of *Lactobacillus* spp. and a progressive decrease in vaginal pH when microablative fractional radiofrequency was used for the treatment of genitourinary syndrome of menopause [93]. Finally, vaginal microbiome transplantation from healthy donors to women suffering from intractable and recurrent bacterial vaginosis can be used to reconstitute vaginal flora towards a *Lactobacillus*-dominated vaginal microbiome [94,95], with potential consequences for UTI incidence.

In addition to diagnostic considerations, the urinary microbiome also has potential implications in the prevention and treatment of UTIs. A recent study by Wolff et al. aimed to appraise the change in the ratio between uropathogens and *Lactobacillus* species within the lower urinary tract in response to orally administered probiotics [96]. This pilot, double-blinded, randomized controlled trial conducted on healthy pre-menopausal female volunteers showed no difference between placebo and probiotic groups regarding the aforementioned ratio; moreover, the probiotic species were never identified in the voided urine, and there were no changes in microbiota diversity between groups [96]. Nevertheless, a study by Thomas-White et al. opened the door to the possibility of reducing the occurrence of post-operative UTI by tackling the depletion of *Lactobacillus* and the enrichment of uropathogens in the bladder prior to surgical procedures with potential probiotic treatments [76]. One such approach may be the application of topical vaginal estrogen with the intent of indirectly increasing or restoring vaginal *Lactobacillus* as a potential treatment approach aimed at improving the urinary bladder microbiome in order to reduce post-operative UTI risk. All of this warrants further reinvestigation through randomized controlled trials.

## 7. Conclusions

The advent of high-throughput sequencing technologies enabled unprecedented insights into the diverse, interrelated, and complex interactions of microorganisms in the human body that influence both healthy and diseased states [1,97]. It seems that the gut, vagina, and urinary bladder represent a trifecta of anatomical sites jointly implicated in the pathogenesis of UTI, with resident microbiota either serving as a potential reservoir of uropathogenic bacteria, or protecting us from their potential to cause UTI (Figure 1). This is why there is also a need to revisit our approach to assessing urinary specimens with mixed microbial growth, which will clearly require a sort of paradigm shift to take into account the increased repertoire of the urinary bacteria, as well as to implement novel diagnostic techniques and algorithms. However, the increasing pace of microbiome characterization in these different niches is still not coupled with translational solutions in our therapeutic and diagnostic armamentarium, which emphasizes the need for devising randomized control trials that will exploit these novel findings.
